# Effects of Fluid Shear Stress on Expression of Smac/DIABLO in Human Umbilical Vein Endothelial Cells^☆^

**DOI:** 10.1016/j.curtheres.2012.11.002

**Published:** 2013-06

**Authors:** Feng Zhang, Le Zhang, Liang-liang Sun, Xiang-lan Meng, Yun Zhao, Xin Jin

**Affiliations:** Department of Pharmacology, School of Medicine, Xiamen University, Xiamen, China

**Keywords:** apoptosis, atherosclerosis, human umbilical vein endothelial cells, shear stress, Smac/DIABLO

## Abstract

**Objective:**

To investigate the molecular mechanisms of laminar shear stress on inhibition of apoptosis in endothelial cells, human umbilical vein endothelial cells (HUVECs) were starved in medium containing 2% fetal bovine serum and 20 dyne/cm^2^ shear stress.

**Methods:**

HUVECs were subjected to shear stress or incubated in a static condition and then Smac/DIABLO expression was quantified by reverse-transcription polymerase chain reaction, real-time PCR, and western blot. The effect of shear stress on the migration of Smac/DIABLO proteins was detected by immunofluorescence microscopy.

**Results:**

Results demonstrated that 20 dyne/cm^2^ shear stress inhibited the expression of Smac/DIABLO at both the mRNA and protein levels in cultured HUVECs. Furthermore, release of Smac/DIABLO from mitochondria was induced by removal of basic fibroblast growth factor and decrease of fetal bovine serum in the medium, whereas shear stress inhibited its release under the same conditions.

**Conclusions:**

These results suggest that down-regulation of Smac/DIABLO may contribute to the potent antiatherosclerotic effect of shear stress by preventing endothelial cells from entering apoptosis.

## Introduction

Shear stress induced by blood flow may play a pivotal role in the induction or prevention of atherosclerosis by affecting endothelial functions. Recent studies have shown that shear stress also inhibits apoptosis of vascular endothelial cells (ECs).^1,2^ In contrast, flow that has low mean shear stress and turbulence is strongly correlated with EC dysfunction, EC apoptosis, and atherosclerosis.^3–5^ In accordance with our previous studies,^6,7^ we found that physiologic shear stress inhibits EC apoptosis partly by activating the human inhibitor of apoptosis protein-2 (HIAP-2) protein and X-linked inhibitor of apoptosis protein (XIAP) protein. The Smac protein, also known as DIABLO, is a recently identified mitochondrial protein and is released from mitochondria together with cytochrome c in the presence of apoptotic stimuli. Smac/DIABLO appears to function as a general inhibitor of inhibitor proteins of apoptosis (IAP) in that it is shown to bind to XIAP, cellular inhibitor of apoptosis 1, cellular inhibitor of apoptosis, Survivin, Livin/melanoma inhibitor of apoptosis, and BRUCE.^8^ It is noteworthy that many studies have shown the importance of Smac/DIABLO pathway in different cell types.^9,10^ However, its role in the endothelial adaptive response to laminar shear stress has, so far, not been elucidated.

Through our study, we show that Smac/DIABLO expression and function are strongly inhibited following exposure of ECs to laminar shear stress. We also analyze the important role that Smac/DIABLO signaling may play in the response of ECs to hemodynamic force.

## Materials and Methods

### Cell culture

Human umbilical vein endothelial cells (HUVECs) were harvested from human umbilical vein using 0.05% trypsin with 0.02% EDTA and suspended in Dulbecco’s modified Eagle’s medium (DMEM) containing 20% fetal bovine serum (FBS), 10 μg/L basic fibroblast growth factor (bFGF), 100 U/mL penicillin, and 100 μg/mL streptomycin sulfate. Cells were plated on gelatin-coated polyester sheets (54 mm × 89 mm; Plastic Suppliers, Columbus, Ohio), at a seeding density of 1∼5 × 10^6^ per sheet. The cells were cultured until they reached confluence. To avoid phenotypic changes caused by passaging, only cells experiencing 3 to 7 passages were used.

**Figure 1:**
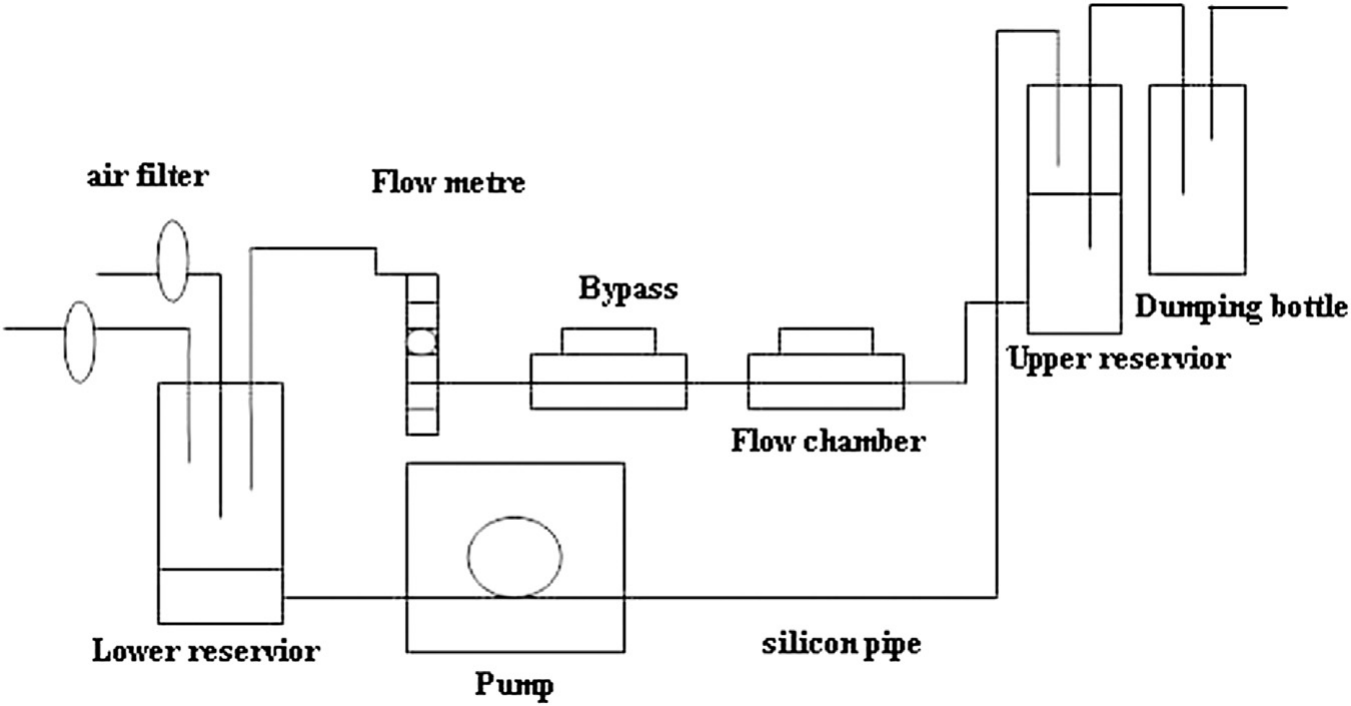
Diagram of flow apparatus. Endothelial cells were cultured on the sheets, and then put into the flow chambers. Different levels of shear stress were produced by adjusting the flow rate.

HUVECs were obtained and cultured as described previously.^6^ These cells were plated on plastic slides or polyester sheets and flow experiments using a parallel-plate flow chamber (Figure 1), which generates a unidirectional laminar shear stress, were performed.^6,7^ Control ECs were maintained in an incubator.

### DNA fragmentation assay

The isolation of fragmented DNA was carried out as follows. Briefly, after culturing for 24 hours and starving in medium containing 2% FBS, cells were treated with varying levels of shear stress for 24 hours. HUVECs were lysed and treated with ribonuclease A, and with proteinase K. The DNA fragments were precipitated with ethanol, resuspended in 50 μL tris(hydroxymethyl)aminomethane and EDTA buffer, and analyzed by electrophoresis.

### Reverse transcription-Polymerase Chain Reaction (RT-PCR) and Real-time PCR

The HUVECs were incubated with 2% FBS DMEM without bFGF for 12 hours, then placed in the shear stress flow chamber. ECs were exposed to 20 dyne/cm^2^ shear stress for 4, 6, 8, and 12 hours, and then cells were harvested for RT-PCR. Total RNA was extracted using Trizol (Invitrogen, Carlsbad, California) according to the manufacturer′s protocol. PCR primers were in accordance with the published sequences as follows: Smac/DIABLO: forward 5’ *GAA GCT GGA AAC CAC TTG GAT GA* 3’, reverse 5’ *TGA ATG TGA TTC CTG GCG GTT A* 3’;*GAPDH*:forward 5’*GCA CCG TCA AGG CTG AGA AC* 3’, reverse 5’*TGG TGA AGA CGC CAG TGG A 3’*. PCR was performed 1 cycle of 95°C for 2.5 minutes, and then run for 33 circles at 94°C for 30 seconds, 58°C for 30 seconds, and 72°C for 45 seconds.

mRNA levels were also quantified by real-time PCR. The amplification mixtures (20 μL) contained 0.5 μL cDNA, 0.5 μL of each primer, and 10 μL SYBR Green PCR Master Mix (Applied Biosystems, Foster City, California). PCR was performed at 50°C for 2 minutes and 95°C for 10 minutes (for AmpliTaq Gold [Invitrogen] activation) then run for 40 cycles at 95°C for 15 seconds and 60°C for 1 minute. The fluorescence data for the SYBR Green dye at each cycle were collected with an ABI PRISM 7300 Sequence Detection System (Applied Biosystems). The cycle threshold values were normalized against the housekeeping gene *GAPDH*.

### Western blot analysis

HUVECs were harvested by scraping, and the cells were pelleted by centrifugation and resuspended in lysis buffer. Proteins were separated on a 10% sodium dodecyl sulfate-polyacrylamide gel and transferred onto a polyvinylidene difluoride membrane. Anti-Smac/DIABLO monoclonal antibody was diluted 1:1,000, antiβ-actin antibody (both Santa Cruz Biotechnology, Santa Cruz, California) was diluted 1:1,000. Immunoblots were detected by enhanced chemiluminescence reaction reagents and signals were quantified by the Quantity One system image program (BioRad, Hercules, California).

### Immunofluorescence microscopy

Cells were exposed to 20 dyn/cm^2^ shear stress with 2% FBS of DMEM for 12 hours and 24 hours, and then 100 nM MitoTracker Red CMXRos (Invitrogen) was added for 20 minutes at 37°C. The cells were washed 3 times with phosphate-buffered saline (PBS), fixed with 4% paraformaldehyde for 20 minutes, permeabilized with 0.2% Triton X-100, obtained from Sigma Chemical, in PBS for 5 minutes, and blocked for 1 hour with 3% bovine-specific antigen (BSA) at 4°C. Cells were then incubated with anti-Smac/DIABLO (1:200 in 3% BSA) at 4°C overnight, washed 3 times with PBS, permeabilized with 0.2% Triton X-100 again, and then incubated with fluorescein isothiocyanate-goat anti-mouse immunoglobulin G (1:100 in 3% BSA) for 60 minutes at 25°C. After 3 more washes with PBS, cells were mounted in 1 drop of Dapi-Fluoromount-G (Southern Biotechnology, Birmingham, Alabama). Finally, the fluorescence was visualized using a laser scanning confocal microscope (Fluoview, Olympus, Japan).

## Results

### Shear stress inhibits the apoptosis of HUVECs

**Figure 2:**
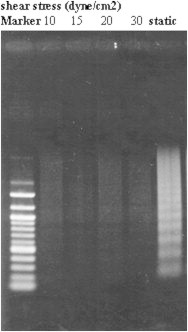
Inhibition of endothelial cell apoptosis by shear stress. After being exposed to shear stress for 24 hours (lanes 2 through 5 show shear stresses of 10, 15, 20, and 30 dyne/cm^2^, respectively), DNA fragmentation of human umbilical vein endothelial cells was analyzed by electrophoresis. Lanes 1 and 6 show a molecular marker and the static control endothelial cells, respectively.

Serum starvation of HUVECs resulted in cell shrinkage, rounding, and membrane blebbing, typical characteristics of apoptotic cells, whereas no such effect was observed with the shear stress-treated cells. Agarose gel electrophoresis revealed a typical ladder pattern of internucleosomal DNA fragmentation in the serum-deprived cells, whereas the shear stress-treated group showed inhibition of serum starvation-stimulated apoptosis (see Figure 2).

### Smac/DIABLO expression in HUVECs

**Figure 3:**
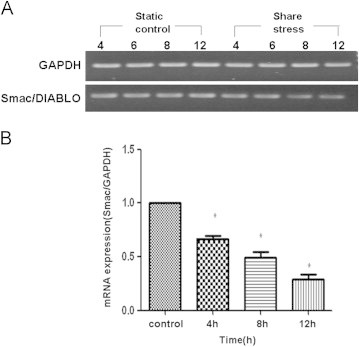
Effect of shear stress on Smac/DIABLO expression in human umbilical vein endothelial cells (20 dyne/cm^2^). RNA samples were collected after 4, 6, and 12 hours of incubation with or without shear stress treatment and Smac/DIABLO expression was quantified by (A) reverse-transcription polymerase chain reaction and (B) real-time polymerase chain reaction. *P < 0.01 versus control.

To examine if shear stress induced expression of Smac/DIABLO mRNA, RT-PCR and real-time PCR were performed. Before the shear stress was imposed, the HUVECs were induced to apoptosis by incubation with 2% FBS DMEM without bFGF for 12 hours. The level of mRNA expression was indicated in terms of the Ct value. The Ct values reflect the cycle number at which the fluorescence generated within a reaction crosses the threshold. ΔCt is the difference in threshold cycles for the target gene and reference gene. In this study, *GAPDH* was used as the reference gene. The Smac/DIABLO mRNA level became lower from 4 hours to 12 hours after shear stress (see Figures 3A and 3B).

### Smac/DIABLO protein migration experiments

To test if Smac/DIABLO protein migrated between the cytoplasm and mitochondria after induction of apoptosis and protection by shear stress, we separated the total protein into cytoplasmic and mitochondrial fractions. Then we used western blotting and immunofluorescence to analyze the results.

**Figure 4:**
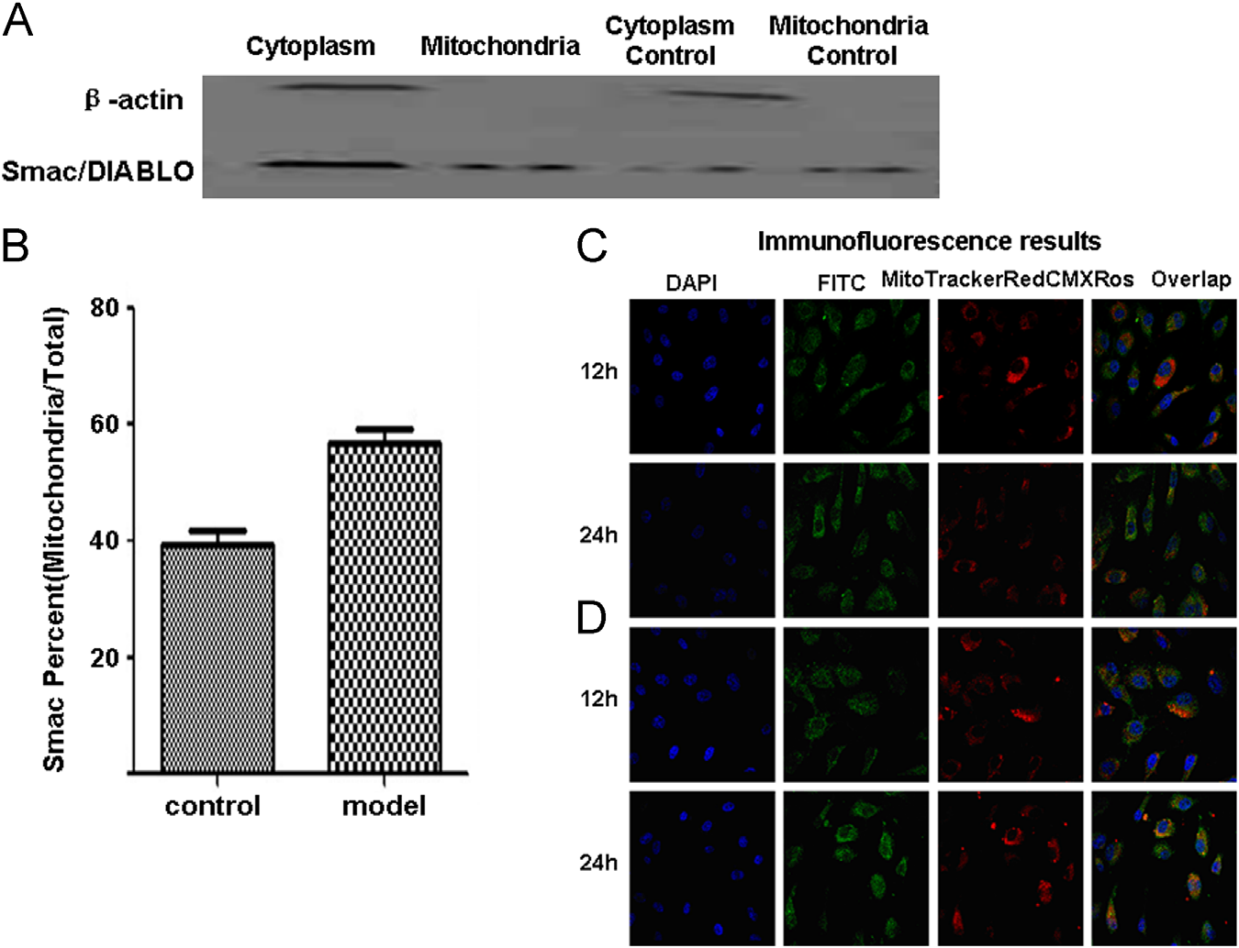
Effect of shear stress on the migration of Smac/DIABLO protein. Human umbilical vein endothelial cells were subjected to shear stress or incubated in a static condition for 24 hours and then western blot analysis was performed to measure the level of Smac/DIABLO protein. The mitochondrial and cytoplasmic proteins were separated. (A) Smac/DIABLO protein was expressed mainly in mitochondria after exposure to 20 dyne/cm^2^ shear stress for 24 hours and (B) protein expression percent at 24 hours. Immunofluorescent staining of Smac/DIABLO in human umbilical vein endothelial cells treated with or without exposure to 20 dyne/cm^2^ shear stress for 12 hours and 24 hours. (C) Cells cultured in the 2% fetal bovine serum and Dulbecco's modified Eagle’s media described previously were used as the static control group and (D) cells experiencing 20 dyne/cm^2^ shear stress are shown in the following images. Smac/DIABLO protein is expressed in both cytoplasm (green) and mitochondria (red). This result is representative of 3 independent experiments. DAPI, 4',6-diamidino-2-phenylindole; FTTC, flourescein isothiocyanate.

Mitochondrial and cytoplasmic proteins were separated by the isolation kit (Applygen, Beijing, China), and the Smac/DIABLO protein levels were measured by western blot. As shown in Figures 4A and 4B, stimulated by 20 dyne/cm^2^ shear stress for 24 hours, the mitochondrial protein was less expressed than the cytoplasmic protein in the static control group. But in the shear stress group, mitochondrial protein expressed more strongly than cytoplasmic protein. The total protein expression of the static control group gradually increased. The total protein expression of the shear stress group, which was exposed to 20 dyne/cm^2^ shear stress, significantly decreased at 24 hours.

For the immunofluorescence analysis, we labeled nuclei with fluorescent 4',6-diamidino-2-phenylindole (blue), Smac/DIABLO with fluorescein isothiocyanate (green), and mitochondria with MitoTracker Red CMXRos (red). Based on analysis of the images detected by fluorescence confocal microscopy (Figures 4C and 4D), Smac/DIABLO protein was gradually released into the cytoplasm from the mitochondria in the static control group after induction due to lack of bFGF and 2% FBS DMEM medium for 12 hours and 24 hours (Figure 4C). In the shear stress group (Figure 4D), the Smac/DIABLO protein was expressed mainly in the mitochondria after shear stress for 12 hours and 24 hours, consistent with the western blotting result. The results indicate that the apoptosis signal made the HUVECs express much more Smac/DIABLO in the absence of the protective effect of shear stress.

## Discussion

Our study demonstrates that Smac/DIABLO mRNA and protein expression as well as release of Smac/DIABLO from mitochondria into the cytoplasm in HUVECs was decreased by shear stress.

Atherosclerotic lesions are preferentially found in areas with low or turbulent shear stress, whereas areas exposed to steady laminar shear stress are protected.^3,4^ A large body of evidence suggests that endothelial apoptosis contributes to the development of atherosclerotic lesions in areas of low or turbulent flow with prevalent occurrence of apoptosis in the downstream part of the plaque.^4,5^ In vitro studies have shown that laminar shear stress at physiologic levels is sufficient to protect HUVECs from cell death.^11,12^ Several mechanisms have been proposed to account for the antiapoptotic effects of laminar shear stress, including up-regulation of antiapoptotic B-cell lymphoma-extra large,^12^ IAPs,^6,7^ and activation of Akt kinase.^13^ The first identified family of endogenous cellular inhibitors of caspases in mammals is the IAP family. IAPs have been characterized over the past several years. The diversity of triggers against which IAPs suppress apoptosis is greater than that observed for any family of apoptotic inhibitor, including the B-cell lymphoma 2 family. The central mechanisms of IAP apoptotic suppression are through direct caspase and procaspase (ie, caspases 3, 7, and 9) inhibition.^14,15^ In a previous report we demonstrated that HIAP-2 and XIAP mRNA and protein are induced by laminar shear stress in EC.^6,7^ The activity of IAPs is modulated by proteins released from the mitochondria, the most extensively characterized examples of which are Smac/DIABLO.^16,17^ Following release from the mitochondria, Smac/DIABLO acts by competitive antagonism of the binding of caspase-3, caspase-7, and caspase-9 to IAPs, particularly XIAP.^16,17^ By reducing the activity of IAPs, Smac/DIABLO is able to amplify the apoptotic signal transmitted by the intrinsic apoptotic pathway. In HUVECs, Smac/DIABLO plays the same proaopoptotic role; that is, Smac/DIABLO releases from mitochondria during apoptosis induced by tumor necrosis factor-related apoptosis-inducing ligand.^9^ Very little is known about the pathophysiologic role of Smac/DIABLO in ECs during shear stress.

In our study we showed that laminar shear stress significantly reduced Smac/DIABLO mRNA level in a time-dependent way from 4 hours to 12 hours, and also decreased the Smac/DIABLO protein expression for 24 hours. Western blotting and immunofluorescence results indicated that shear stress inhibited Smac/DIABLO release from mitochondria to the cytoplasm. Further investigation is still required to determine the direct effect of Smac/DIABLO on ECs when exposed to laminar shear stress. Because inhibition of IAPs promotes apoptosis, it is reasonable to assume that the decreased expression of Smac/DIABLO may inhibit apoptosis in ECs when exposing to laminar shear stress.

### Future Perspectives

These data provide an explanation for the inhibitory effect of shear stress on apoptosis. Further studies to define the upstream and downstream molecular events involved in shear stress-induced Smac/DIABLO expression will deepen our understanding of the mechanisms by which shear stress regulates endothelial functions. Laminar shear stress-induced inhibition of Smac/DIABLO produced by ECs is likely to play an important role in the formation and development of atherosclerosis.

## Conclusions

Our study provides the first evidence that physiologic levels of laminar shear stress reduce Smac/DIABLO expression and release into cytoplasm in HUVECs in vitro. Notably, we found that 20 dyne/cm^2^ shear stress inhibited the expression of Smac/DIABLO, release of Smac/DIABLO from mitochondria was induced by removal of bFGF and decrease of FBS, and shear stress inhibits the release of Smac/DIABLO release under the same conditions.

## Conflicts of Interest

The authors have indicated that they have no conflict of interest regarding the content of this article.
